# Nitrofurantoin-associated Acute Pulmonary Toxicity Mimicking Severe Sepsis with Significantly Elevated Procalcitonin

**DOI:** 10.7759/cureus.5516

**Published:** 2019-08-29

**Authors:** Tahir Muhammad Abdullah Khan, Yusra Ansari, Abdul Hasan Siddiqui, Hall Matthew, Faraz Siddiqui

**Affiliations:** 1 Internal Medicine, Marshfield Medical Center, Marshfield, USA; 2 Internal Medicine, Rawalpindi Medical College, Rawalpindi, PAK; 3 Pulmonary and Critical Care Medicine, Staten Island University Hospital / Northwell Health, Staten Island, USA; 4 Infectious Disease, Marshfield Medical Center, Marshfield, USA; 5 Pulmonary and Critical Care Medicine, Robert Packer Hospital, Sayre, USA

**Keywords:** nitrofurantoin, pulmonary toxicity, sepsis, procalcitonin

## Abstract

Nitrofurantoin is a commonly used treatment for urinary tract infections with a risk for pulmonary toxicity. We report a case of a 48-year-old woman on a prophylactic regimen of nitrofurantoin who exhibited classic signs of bacterial sepsis including elevated procalcitonin (PCL) and C-reactive protein (CRP) levels two days post-nephrolithotripsy. The microbial analysis did not reveal an infectious cause for the initial symptoms and, subsequently, the patient developed a dry cough, fever, chills, and transient hypoxemia requiring supplemental oxygen. Pulmonary imaging revealed significant abnormal features inconsistent with the patient’s symptoms which indicated an inflammatory/immune reaction to nitrofurantoin. Treatment discontinuation improved the patient’s symptoms and reduced PCL and CRP levels to within normal limits. A high index of suspicion for nitrofurantoin-associated pulmonary toxicity is warranted for patients on a regimen of nitrofurantoin who exhibit severe pulmonary symptoms and elevated PCL and CRP levels with no corresponding infection.

## Introduction

Nitrofurantoin is used to treat uncomplicated urinary tract infection, and its use can lead to acute, subacute, or chronic pulmonary toxicity [1]. Nitrofurantoin-associated pulmonary toxicity can manifest as cough, mild fever, shortness of breath, and pulmonary infiltrates on clinical imaging [2-7]. We report a hyperacute presentation of nitrofurantoin-induced toxicity initiated within two days of nitrofurantoin use manifesting as a severe systemic inflammatory response with fever, shaking, chills, lethargy, leukocytosis, significantly elevated C-reactive protein (CRP) and procalcitonin (PCL) levels mimicking severe sepsis of bacterial origin though no infective cause for these symptoms was found. The patient further developed mild transient hypoxemic respiratory failure with dry cough that was associated with bilateral multifocal pulmonary infiltrates and bilateral small pleural effusions with basilar atelectasis. The clinical condition of the patient spontaneously improved by cessation of nitrofurantoin use, and inflammatory markers rapidly trended downward with no additional complications post-nitrofurantoin discontinuation. This case report highlights a unique hyperacute presentation of nitrofurantoin-induced pulmonary toxicity characterized by a severe inflammatory response with significantly elevated PCL level (25.5 ng/mL), a marker which is generally indicative of bacterial sepsis [8,9].

## Case presentation

A 48-year-old female with history of hypertension, obesity, polycystic ovarian syndrome, and right-sided nephrolithiasis experienced right-flank severe colicky pain with nausea and non-bloody vomiting within 24 hours post-urology intervention of right ureteroscopy with laser lithotripsy, stone extraction, and double-J stent placement. The patient’s symptoms were secondary to the displacement of a ureteral stent that resulted in spontaneous expulsion of the stent with subsequent mild improvement of symptoms. However, the patient then developed shaking, chills, and a fever of 39.1°C (102.4°F) associated with lethargy and weakness. The patient denied urinary, respiratory, or other symptoms, and a chart review indicated the patient had been discharged home post-surgery on a prophylactic regimen of 100 mg nitrofurantoin administered twice daily.

**Figure 1:**
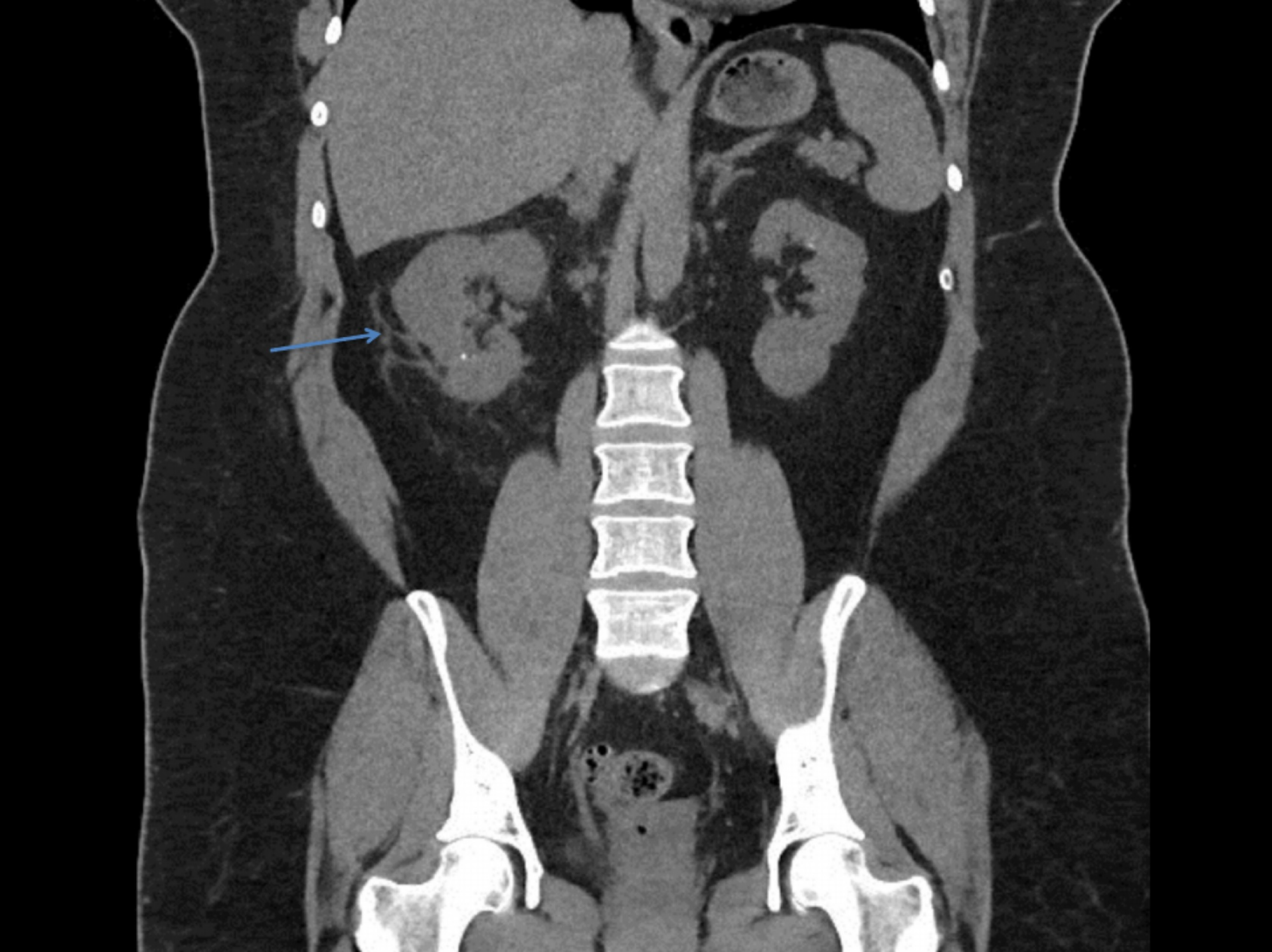
Computed tomography imaging of the abdomen and pelvis without contrast Computed tomography imaging of the abdomen and pelvis. Note the multiple small calculi in both kidneys with mild hydronephrosis, ureteral edema, and stranding of the right kidney and collecting system (arrow).

On examination, the patient appeared lethargic and had dry oral mucous membranes. Abdominal exam revealed a soft, non-distended abdomen with mild right flank discomfort. Chest auscultation was clear except for bibasilar mild crackles. Vitals were normal except for mild tachycardia. Lab work showed leukocytosis of 19,000 cells/µL (normal: 4,000-11,000/µL) with elevated lactic acid of 2.5 mmol/L (normal lactic acid: <2.0 mmol/L), serum creatinine (SCr) of 1.3 mg/dL (baseline SCr: 1.0 mg/dL), and elevated blood urea nitrogen (BUN) of 26 mg/dL. In addition, PCL and CRP levels were elevated to 25.5 ng/mL and 39.3 mg/dL, respectively, indicating a bacterial infection. Urinalysis was unremarkable for infection or hematuria. To evaluate flank pain, a computed tomography (CT scan) of the abdomen and pelvis without contrast was performed which revealed multiple small calculi in both kidneys with mild right hydronephrosis, ureteral edema, and stranding of the right kidney and collecting system suggesting reactionary changes due to recent instrumentation versus possible infection (Figure 1). Since the patient was on a prophylactic nitrofurantoin regimen, we presumed that urinalysis with no pyuria was secondary to the use of nitrofurantoin, and due to recent urinary tract intervention, right kidney and ureteral inflammation on CT scan, a urinary source of infection was highly suspected as contributing to the clinical condition. After taking urine cultures and blood cultures, the patient was initiated on an empiric regimen of intravenous piperacillin-tazobactam at 3.375 gm every 8 hours, and nitrofurantoin was discontinued.

**Figure 2:**
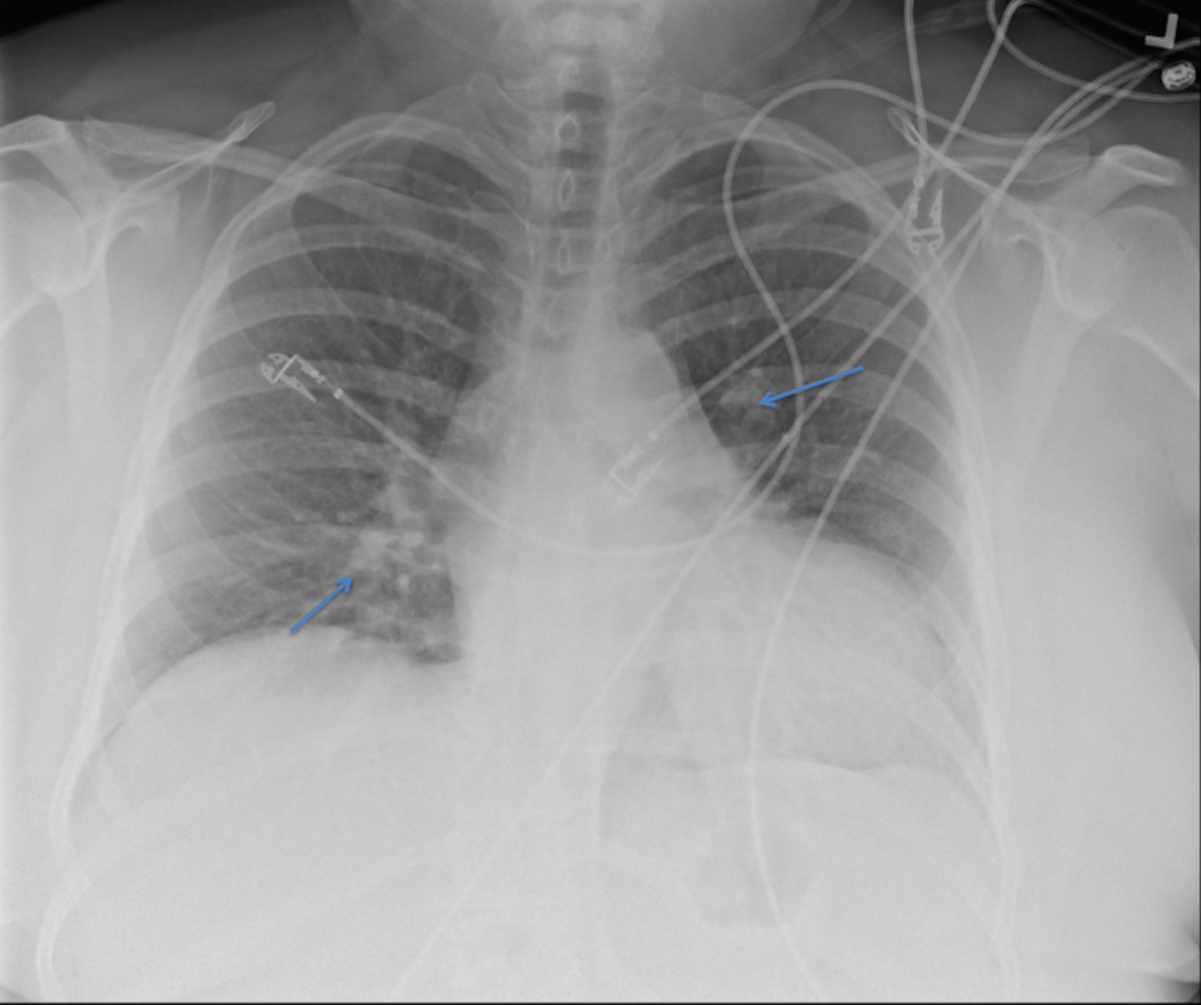
Chest X-ray Chest X-ray to identify pulmonary abnormalities. Note the asymmetric left suprahilar density and right infrahilar density in this image (arrows).

Within 24 hours of admission, the patient developed mild dry cough, a fever of 38.89°C (102°F), and chills associated with transient hypoxia with a pulse oximetry desaturation to 86% requiring 1 L nasal cannula oxygen to maintain normal oxygenation. A chest radiograph revealed an asymmetric left suprahilar density and right infrahilar density consistent with atelectasis, aspiration, or multifocal pneumonia (Figure 2).

**Figure 3:**
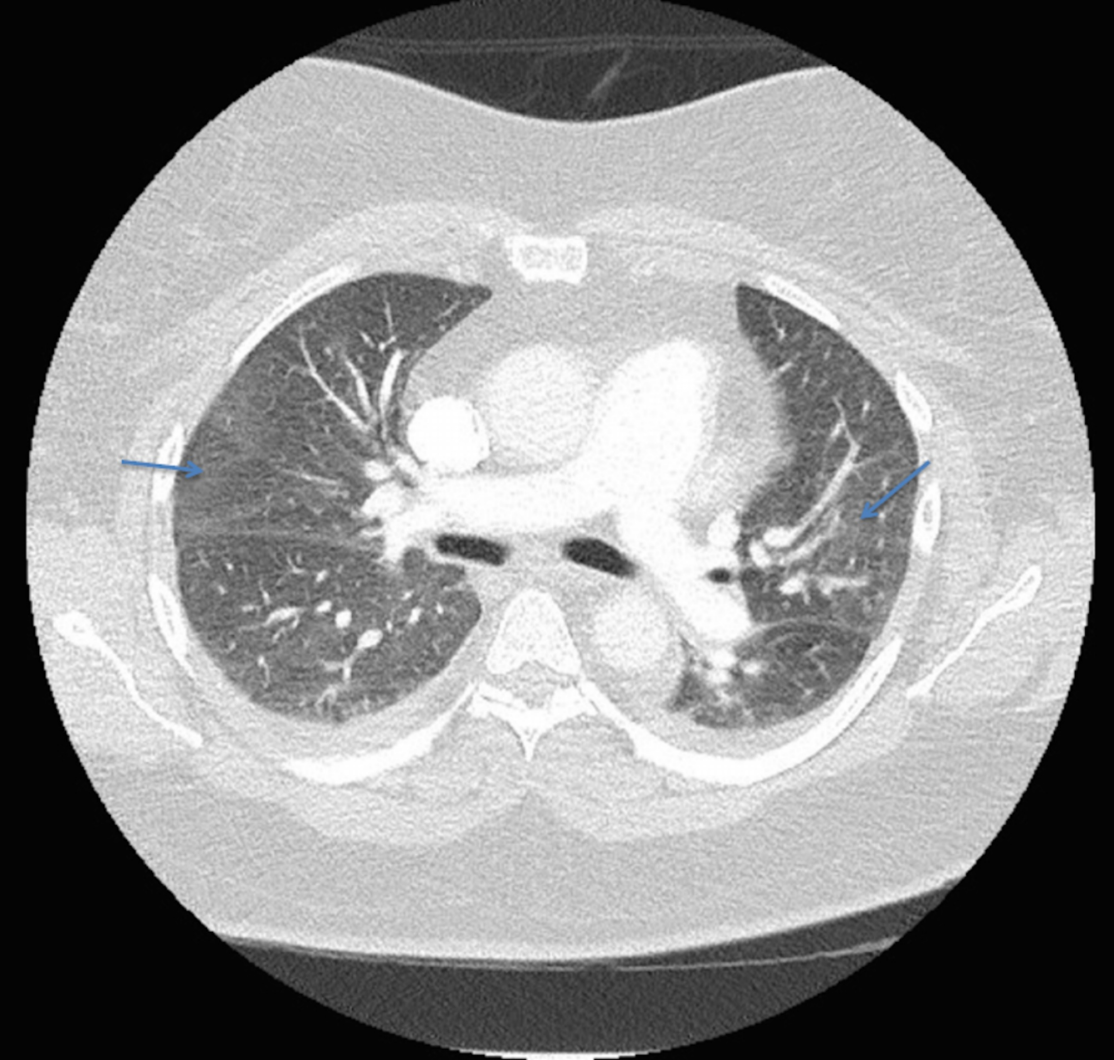
Computed tomography of chest with contrast Computed tomographic imaging of the chest revealed no pulmonary embolism, but multifocal ground-glass opacities were observed (arrows).

Comprehensive respiratory viral panel, urine Legionella, and streptococcal pneumonia antigen were negative. Sputum cultures could not be taken because of nonproductive cough. Preliminary urine cultures and blood cultures revealed no growth. Due to atypical presentation of pulmonary symptoms, follow-up CT angiography of the chest revealed no evidence of pulmonary emboli. The ground glass opacities were present throughout both lungs with small bilateral pleural effusions and adjacent bibasilar atelectasis (Figure 3).

Pulmonary imaging findings were out of proportion to the clinical condition of the patient. Urology and infectious disease physicians evaluated the patient, and it was considered that nitrofurantoin use was contributing to acute onset of bilateral multifocal pulmonary infiltrates leading to mild transient cough and transient pulse oximetry desaturation associated with physiological systemic inflammatory stress response as evidenced by fever, leukocytosis, and significantly elevated PCL and CRP. After the third day of hospitalization, the piperacillin-tazobactam regimen was discontinued, and the patient was observed off all antibiotics. The patient continued to improve without antibiotics and did not require supplemental oxygen with resolution of symptoms within the next three days. We determined that inflammatory findings present in imaging of the right kidney and collecting system were likely secondary to recent urological instrumentation with laser lithotripsy while the transient right-sided flank pain was probably secondary to displacement of the ureteral stent leading to ureteral colic. Serum inflammatory markers continued to improve without antibiotics after cessation of nitrofurantoin use with decreases in serum PCL and CRP levels from admission to discharge day specifically 25.5 ng/mL to 2.1 ng/mL and 39.3 mg/dL to 14.5 mg/dL, respectively. The patient was discharged after six days of hospitalization, and it was strongly recommended the patient avoid use of nitrofurantoin in the future to prevent recurrence of pulmonary toxicity. The patient continues to have a stable clinical condition after discharge and no complications have been reported since then.

## Discussion

Nitrofurantoin is used to treat acute uncomplicated cystitis and as a prophylaxis for recurrent uncomplicated urinary tract infections [1]. Beers criteria recommend against the use of nitrofurantoin in patients 65 years and older due to its potential side effects of pulmonary toxicity, hepatotoxicity, and peripheral neuropathy [10]. Nitrofurantoin toxicity can present as acute, subacute, or chronic pulmonary reaction with a variety of clinical manifestations, but in our patient, it presented as severe sepsis with pulmonary infiltrates [2,4,6,7]. Sovijärvi et al. reported that acute pulmonary toxicity is more common than subacute or chronic toxicity with onset of acute pulmonary reaction usually noticed after a mean of 8.7 days after the initiation of nitrofurantoin though onset can be as early as within 24 to 48 hours of nitrofurantoin use as seen in our patient [2]. Subacute pulmonary reactions usually occur after one month of nitrofurantoin use; however, chronic nitrofurantoin-induced pulmonary toxicity usually manifests after a median interval of six to 23 months when nitrofurantoin was used as prophylaxis for recurrent urinary tract infection [2,5-7].

Nitrofurantoin pulmonary toxicity is more commonly observed in women compared to men though the high prevalence observed in women is probably secondary to a greater susceptibility of women to recurrent urinary tract infections requiring antibiotic therapy [2,3,5,6,11]. According to the Swedish Adverse Drug Reaction Committee Registry, 85% of patients diagnosed with nitrofurantoin-induced acute or chronic pulmonary toxicity were the women with a median age of 59 years for acute reaction versus 68 years of age for chronic pulmonary toxicity [3,12].

Patients with nitrofurantoin-induced pulmonary toxicity usually present with fever, cough, dyspnea, eosinophilia in blood, and bilateral lung infiltrates seen on chest images [2,4-7]. The most common imaging findings are bilateral infiltrates seen on chest radiography and bilateral ground-glass opacities detected on CT scans of the chest, but in some cases, subpleural irregular linear opacities and patchy consolidations can be observed [2,3,5-7].

A majority of patients with nitrofurantoin-associated pulmonary toxicity show rapid improvement with the cessation of nitrofurantoin [2]. However, in patients with no improvement with cessation of nitrofurantoin or with questionable diagnosis, a bronchoalveolar lavage (BAL) analysis may be considered to evaluate for infection, lymphangitic carcinomatosis, or alveolar hemorrhage. BAL in patients with nitrofurantoin-induced toxicity usually have eosinophilia, neutrophilia, and lymphocytosis [13,14]. It is important to note that acute eosinophilic pneumonia associated with acute respiratory distress syndrome due to nitrofurantoin use has been reported in the literature and should be considered a possible diagnosis for patients with a recent history of nitrofurantoin use, as these patients respond well to the cessation of culprit drug and with corticosteroid therapy [13].

The pulmonary biopsy is usually not needed in patients with nitrofurantoin-induced toxicity though it may be considered when the patient does not show improvement after discontinuation of nitrofurantoin, and there is a high index of suspicion for alternative diagnoses. In an acute reaction, a pulmonary biopsy usually reveals an acute reaction with eosinophils, interstitial inflammation, vasculitis, and alveolar exudates [2]. However, in cases of subacute and chronic pulmonary reaction that usually develop after at least one and six months of treatment, respectively, lung biopsy reveals nonspecific lung tissue inflammation, interstitial fibrosis, and capillary sclerosis [2,5].

Most patients with acute, subacute, and chronic lung injury respond well to cessation of nitrofurantoin therapy resulting in spontaneous clinical recovery; however, those with severe pulmonary toxicity may need systemic corticosteroid therapy [2,5,7,13]. Patients with acute pulmonary toxicity usually recover within 15 days of discontinuing nitrofurantoin use, but in patients with chronic pulmonary toxicity, recovery may take longer, and the signs of pulmonary fibrosis may persist for a longer time in more than 50% of patients [2,3]. Patients with both acute and chronic nitrofurantoin-induced pulmonary toxicity generally have a good prognosis including those patients with lung biopsy or radiographic evidence of chronic interstitial lung disease [2,5,15]. Patients with nitrofurantoin-induced interstitial lung disease with severely distorted bronchial architecture and honeycombing observed on imaging may benefit from a transbronchial biopsy to assess the reversibility of pulmonary lesions [16]. Findings of acute or subacute interstitial pneumonitis on lung biopsy usually suggest a good prognosis, and these patients usually respond well to prednisone therapy in addition to discontinuation of nitrofurantoin [16].

Despite a generally good prognosis, fatalities have been reported in the literature. According to the medicine and healthcare products regulatory authority report, from 1963 until 2010, 11 out of 392 patients affected with nitrofurantoin-associated lung injury were reported to have a fatal outcome [6]. Therefore, caution should be exercised when prescribing a nitrofurantoin regimen, and patients with severe symptoms of hypoxia and multifocal lung infiltrates on radiography should be hospitalized for close monitoring and symptom management.

Our patient’s presentation of nitrofurantoin-induced toxicity was unique in that it mimicked severe bacterial sepsis including tachycardia, lethargy, high-grade fever, chills, mild nonproductive cough with dyspnea, and hypoxia associated with elevated PCL and CRP levels in addition to leukocytosis without eosinophilia. PCL is considered a more specific marker for bacterial infection compared to other inflammatory markers such as serum leukocyte count, CRP, and erythrocyte sedimentation rate though recent work indicates comparable diagnostic performance with PCL and CRP as sepsis markers [8,9]. However, this is the first reported case of acute pulmonary toxicity due to nitrofurantoin mimicking bacterial infection with significant elevations in PCL. Nevertheless, such a diagnosis warrants a careful evaluation of the patient including infectious disease work-up to exclude the possibility of infectious cause as a contributing factor to the patient's symptomatology, and it is important to note that absence of eosinophilia usually observed in nitrofurantoin-induced pulmonary injury does not rule out a diagnosis of nitrofurantoin-induced pulmonary toxicity.

## Conclusions

Nitrofurantoin-induced pulmonary toxicity can occur as early as within 24 to 48 hours of initiation of drug exposure, and recent medications use by the patient should be explored at the time of presentation for all patients exhibiting respiratory symptoms and sepsis with no identifiable infectious cause. Though elevated PCL is a marker for bacterial sepsis, it can be elevated in nitrofurantoin-pulmonary toxicity without bacterial infection. Early cessation of nitrofurantoin therapy in patients with hyperacute pulmonary toxicity carries a good prognosis and results in early resolution of symptoms without the need for systemic steroids; patients should be recommended to not use nitrofurantoin in the future to avoid toxicity recurrence.

Due to the higher prevalence of nitrofurantoin-induced pulmonary toxicity, physicians should be cautious in prescribing nitrofurantoin, and patients should be informed about the possibility of pulmonary toxicity along with other adverse effects at the time of prescription. Providers should also continue long-term surveillance of patients on a long-term nitrofurantoin regimen to ensure early detection of toxicity and to discontinue nitrofurantoin promptly.
